# The Prediction of Cardiac Events Using Contemporary Risk Prediction Models after Radiation Therapy for Head and Neck Cancer

**DOI:** 10.3390/cancers14153651

**Published:** 2022-07-27

**Authors:** Raza M. Alvi, Thiago Quinaglia, Aferdita Spahillari, Giselle A. Suero-Abreu, Malek Z. O. Hassan, Carlos Gongora, Hannah K. Gilman, Sofia Nikolaidou, Supraja Sama, Lori J. Wirth, Annie W. Chan, Daniel Addison, Tomas G. Neilan

**Affiliations:** 1 Department of Medicine, University of Massachusetts Medical School, Baystate Medical Center, Springfield, MA 01199, USA; alvinmed@gmail.com; 2 Cardiovascular Imaging Research Center (CIRC), Department of Radiology and Division of Cardiology, Massachusetts General Hospital, Harvard Medical School, Boston, MA 02114, USA; tquinaglia@mgh.harvard.edu (T.Q.); malekabusarhan@gmail.com (M.Z.O.H.); cgongora@mgh.harvard.edu (C.G.); hkgilman@mgh.harvard.edu (H.K.G.); snikolaidou@mgh.harvard.edu (S.N.); ssama@mgh.harvard.edu (S.S.); daniel.addison@osumc.edu (D.A.); 3 Division of Cardiology, Department of Medicine, Massachusetts General Hospital, Harvard Medical School, Boston, MA 02114, USA; aspahillari@partners.org (A.S.); gsueroabreu@mgh.harvard.edu (G.A.S.-A.); 4 Division of Oncology, Department of Medicine, Massachusetts General Hospital, Harvard Medical School, Boston, MA 02114, USA; lwirth@mgh.harvard.edu; 5 Department of Radiation Oncology, Massachusetts General Hospital, Harvard Medical School, Boston, MA 02114, USA; awchan@mgh.harvard.edu; 6 Cardio-Oncology Program, Division of Cardiology, Department of Medicine, Massachusetts General Hospital, Harvard Medical School, Boston, MA 02114, USA; 7 Cardio-Oncology Program, Division of Cardiology, The Ohio State University Medical Center, Columbus, OH 43210, USA

**Keywords:** head and neck cancer, radiation therapy, statins, ASCVD score, Framingham score, USPSTF

## Abstract

**Simple Summary:**

Radiation therapy is associated with an increased risk for atherosclerotic cardiovascular disease (ASCVD). Contemporary risk prediction models accurately predict ASCVD in general populations. Whether these models adequately capture ASCVD risk after radiation therapy (RT) is unknown. Our data show that these standard risk scores do not reliably differentiate between those who will and those who will not have an ASCVD event after RT and underestimate the risk for ASCVD among patients receiving RT for HNCA.

**Abstract:**

This study aims to evaluate the efficacy of the Pooled Cohort Equation (PCE), U.S. Preventative Services Task Force (USPSTF), and Framingham Risk Score (FRS) models in predicting ASCVD events among patients receiving radiation therapy (RT) for head and neck cancer (HNCA). From a large cohort of HNCA patients treated with RT, ASCVD events were adjudicated. Observed vs. predicted ASCVD events were compared. We compared rates by statin eligibility status. Regression models and survival analysis were used to identify the relationship between predicted risk and post-RT outcomes. Among the 723 identified patients, 274 (38%) were statin-eligible based on USPSTF criteria, 359 (49%) based on PCE, and 234 (32%) based on FRS. During follow-up, 17% developed an ASCVD, with an event rate of 27 per 1000 person-years, 68% higher than predicted (RR 1.68 (95% CI: 1.02, 2.12), *p* < 0.001). In multivariable regression, there was no difference in event rates by statin eligibility status (*p* > 0.05). Post-RT, the observed event rate was higher than the predicted ASCVD risk across all grades of predicted risk (*p* < 0.05) and the observed risk of an ASCVD event was high even among patients predicted to have a low risk of ASCVD. In conclusion, current ASCVD risk calculators significantly underestimate the risk for ASCVD among patients receiving RT for HNCA.

## 1. Introduction

The use of radiation therapy (RT) to treat cancer has increased [1,2]. Ionizing radiation exposure to the heart and vasculature is associated with an increased risk of atherosclerotic cardiovascular (ASCVD) events among long-term survivors of multiple cancer types [3,4,5,6,7,8,9]. The benefits of statin therapy for patients at risk for vascular events is well established [10,11]. This is particularly true in populations in which traditional cardiovascular (CV) risk may be elevated, including those with cancer [12,13]. The ACC/AHA pooled cohort equations (PCE), the Framingham Risk Score (FRS) models, and the United States Preventative Services Taskforce (USPSTF) recommendations are used to predict ASCVD risk and to provide guidance on the initiation of statin therapy for primary prevention of ASCVD [11,14,15]. These models are standard risk prediction models for ASCVD events among the general population [11,14,15]. The accuracy of these models for risk prediction of ASCVD among patients receiving RT involving the vasculature is unclear. Therefore, our aim was to evaluate the efficacy of the PCE and FRS models and of the USPSTF recommendations in predicting ASCVD events among patients receiving RT. We studied patients with head and neck cancers (HNCA), as this represents the sixth most common type of cancer worldwide, with nearly 600,000 persons diagnosed each year, with a particular increase among younger patients. The increased incidence in young patients is due to the shift in risk factors from smoking-related cancer to human papilloma virus–related cancer [16,17]. Additionally, RT is a standard primary treatment for HNCAs, where overall survival rates have improved.

## 2. Materials and Methods

This was a retrospective study. The total cohort included 1011 consecutive patients receiving radiation therapy for HNCA from 2001–2012 at Massachusetts General Hospital, Boston, Massachusetts. From these patients, 723 non-statin treated patients were identified and formed the final cohort for the main analysis. The study was approved by the hospital’s institutional review board. The requirement for written informed consent was waived due to the study design.

### 2.1. Covariates of Interest

Data on covariates including age, sex, race, and body mass index (BMI) were extracted retrospectively from the electronic medical records. Pre-existing CV disease and risk factors including hypertension, dyslipidemia, diabetes mellitus, tobacco use, coronary artery disease (CAD), stroke, cardiomyopathy, heart failure (HF), and atrial fibrillation or flutter were recorded. Information on cardiovascular and chemotherapy medications was also obtained via chart review. Statin eligibility was determined based on ACC/AHA PCE, USPSTF, and the Framingham risk scores [11,14,15]. The USPSTF recommendations for a statin for primary prevention are as follows: For adults aged 40–75 yrs. with no history of CVD, 1 or higher CVD risk factors, and a calculated 10-year CVD event risk of 10% or higher. The PCE approach recommends a statin for primary prevention for the following: individuals with primary elevations of LDL > 190 mg/dL, individuals 40 to 75 yrs. of age with diabetes and 70 to 189 mg/dL without clinical ASCVD, and individuals without clinical ASCVD or diabetes who are 40 to 75 yrs. of age with LDL 70 to 189 mg/dL and a 10-yr ASCVD risk of 7.5% or higher. The FRS model recommends a statin for primary prevention for the following: individuals with a FRS of 20% or higher, individuals with a FRS of 10% to 19% with LDL-C 3.5 mmol/L or higher, individuals with a LDL-C less than 3.5 mmol/L with non-HDL 4.3 mmol/L or higher or men 50 yrs. of age or older and women 60 yrs. of age or older with a 1-risk factor.

### 2.2. Outcome of Interest

Our outcome of interest was the occurrence an ASCVD event, defined as the composite of incident non-fatal myocardial infarction (MI), coronary heart disease (CHD), stroke, and cardiovascular death. When a patient had multiple ASCVD events, the date of the earliest event was defined as the incident ASCVD event. All subject charts were individually reviewed for incident ASCVD events. All outcome events were adjudicated using standard definitions and were confirmed by review of the EHR by the study team blinded to cancer, cardiac, medication, and RT variables. The source of the standard ASCVD risk calculator was the ACC/AHA ASCVD risk estimator tool (https://tools.acc.org/ldl/ascvd_risk_estimator/index.html#!/calulate/estimator/ (accessed on 30 April 2017)).

### 2.3. Statistical Analysis

Descriptive statistics were used to summarize patient characteristics, wherein continuous variables were presented as mean and SD or median (interquartile range, IQR), as appropriate, based on normality, and categorical variables were presented as percentages. Continuous data were compared with the use of unpaired Student *t*-tests or Wilcoxon rank sum tests, as appropriate. Categorical data were compared using the chi-square or the Fisher exact test. The association between statin eligibility and events was determined using unadjusted and adjusted Cox proportional hazard models (for presence or absence of an ASCVD event as binary variable) for the calculation of hazard ratios. We used Fine and Grey models for competing risk analysis. To calculate the incidence rate ratio (IRR), first, we tested our data for dispersion by calculating the mean and variance of the count outcome of the ratio of variance where mean = 1.1, which is close to “1”. We further confirmed by looking at the ratio of value of the deviance to the degree of freedom in the goodness of the fit model of our analysis which was also close to 1, indicating that our count outcome was not over- or under-dispersed. Therefore, we used Poisson regression analysis (incorporating all ACSVD events outcomes as count variable) for the calculation of IRRs. Time-to-event analysis survival analysis (Kaplan–Meier) curves and the log-rank test were used to compare the event rates between statin eligible and statin non-eligible groups across risk prediction models. Statistical significance was defined using a *p*-value ≤ 0.05. The rate of events per 1000 person-years were calculated by standard formula. Statistical analyses were performed using SPSS software version 24 (IBM Corp., Armonk, NY, USA).

## 3. Results

### 3.1. Baseline Patient Characteristics

Overall, from 1011 patients receiving RT for HNCA, 288 patients were on statin treatment either before or after RT, leaving 723 non-statin-treated patients who formed the final cohort. Of these 723, 70% were males, the median age was 60 years, the mean BMI was 27 kg/m^2^, 21% had diabetes mellius, 44% had hypertension, 4% had heart failure, 25% were prescribed aspirin, and 578 (71.5%) received chemotherapy. Of the 723 patients not prescribed statin treatment, 274 (38%) were statin eligible based on USPSTF criteria, 359 (49%) were statin eligible based on PCE, and 234 (32%) were statin eligible based on FRS. As expected, statin eligible patients included more patients with diabetes, hypertension and vascular disease. Otherwise, there was no difference in the remaining baseline characteristics between the statin eligible and statin non-eligible patients (Table 1, Table 2 and Table 3).

### 3.2. Comparison of Event Rates between the Statin Eligible and Statin Non-Eligible Groups

**Figure 1:**
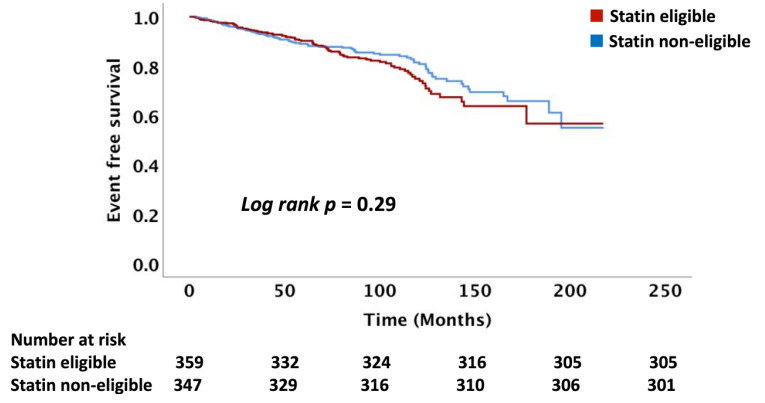
This figure demonstrates the Kaplan–Meier curves comparing the ASCVD events between the statin eligible vs. non-eligible groups based on the ACC/AHA/PCE criteria. T0 = radiation therapy treatment. ASCVD = atherosclerotic cardiovascular disease.

**Figure 2:**
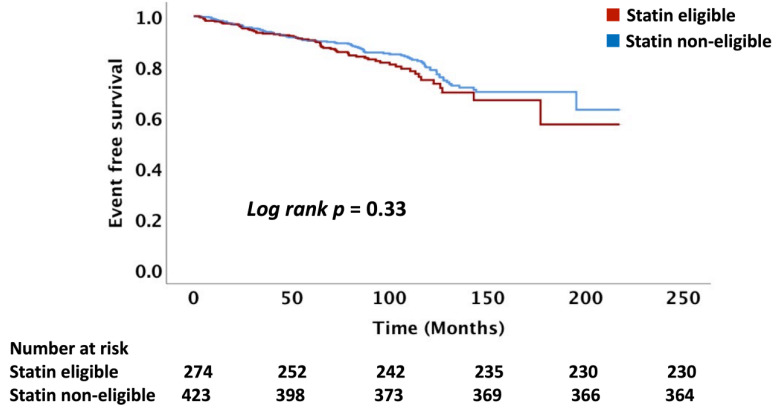
This figure demonstrates the Kaplan–Meier curves comparing the ASCVD events between the statin eligible vs. non-eligible groups based on the USPSTF criteria. T0 = Radiation therapy treatment. ASCVD = atherosclerotic cardiovascular disease.

**Figure 3:**
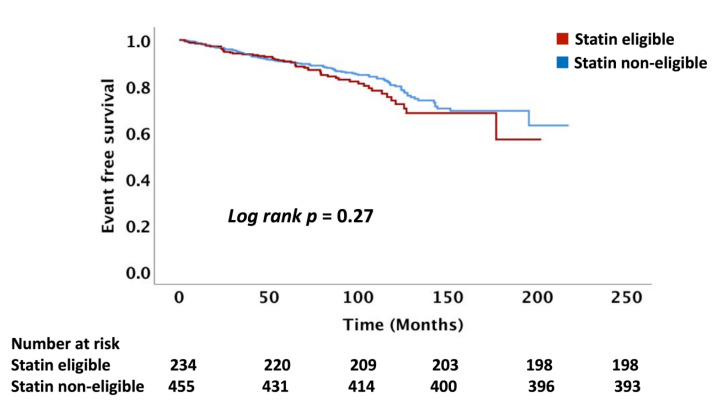
This figure demonstrates the Kaplan–Meier curves comparing the ASCVD events between the statin eligible vs. non-eligible groups based on the Framingham criteria. T0 = radiation therapy treatment. ASCVD = atherosclerotic cardiovascular disease.

**Figure 4:**
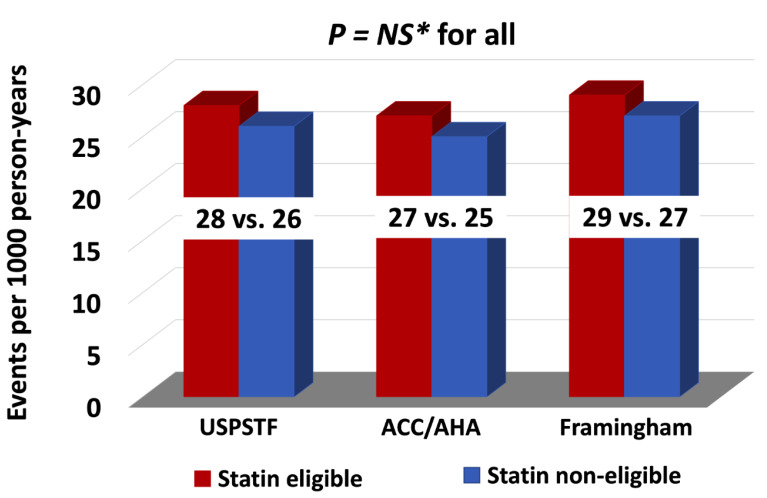
This figure shows bar graphs comparing the event rates between the statin eligible vs. statin non-eligible groups based on USPSTF, ACC/AHA, and FRS; and reduced discrimatory predictive power for ASCVD-risk following radiothearpy. ASCVD = Atherosclerotic Cardiovascular Disease. * NS = Statistically nonsignificant (>0.05).

The follow-up time of the cohort was a median of 6.6 (IQR: 4.3, 9.0) years. Overall, 17% of the cohort of 723 patients not on statin treatment had an ASCVD event with an event rate of 27 per 1000 person-years. We compared the number of ASCVD events and event rates between the statin eligible and statin non-eligible HNCA patients treated with RT as determined using the USPSTF, PCE, and FRS equations. There was no difference in ASCVD event rates between RT-treated HNCA patients when comparing statin eligible vs. non-eligible groups: USPSTF (16 vs. 14%, *p* = 0.41), PCE (15 vs. 13.5%, *p* = 0.36), and FRS (15.5 vs. 13.7%, *p* = 0.43), respectively (Table 4, Figure 1, Figure 2 and Figure 3). Similarly, ASCVD event rates, per 1000 person-years, were similar between the statin eligible vs. non-eligible patients with HNCA treated with RT: USPSTF (28 vs. 26, *p* = 0.35, PCE (27 vs. 25, *p* = 0.32), and FRS (29 vs. 27, *p* = 0.46) (Table 4, Figure 4).

In Cox proportional hazard regression modeling, there was also no difference in the observed ASCVD risk based on statin eligibility or non-eligibility, using the USPSTF, PCE, or FRS (*p* = NS). Specifically, with any of the USPSTF (HR = 1.36, 95% CI (0.82, 1.62), *p* = 0.26), PCE (HR = 1.28, 95% CI (0.86, 1.72), *p* = 0.32), or FRS (HR = 1.17, 95% CI (0.76, 1.58), *p* = 0.42 (Table 5), statin eligibility or non-eligibility was associated with similar risks for ASCVD.

Similar results were obtained with a competing risk model (Fine–Grey Model); USPSTF (HR = 1.31, 95% CI (0.69, 1.52), *p* = 0.27), PCE (HR = 1.20, 95% CI (0.72, 1.64), *p* = 0.31), or FRS (HR = 1.12, 95% CI (0.68, 1.48), *p* = 0.38, and on Poisson regression analysis using all ASCVD events (outcome variable) as count variable: USPSTF: IRR = 1.15, 95% CI (0.84, 1.65), *p* = 0.36, PCE: IRR= 1.17, 95% CI (0.87, 1.57), *p* = 0.38, and FRS: IRR = 1.16, 95% CI (0.74, 1.87), *p* = 0.47. (Table 6 and Table 7). Furthermore, we checked for a correlation between cancer-related deaths and ASCVD events with and without radiation therapy. The Pearson Correlation of cancer-related deaths alone with ASCVD events was very weak (−0.21).

**Figure 5:**
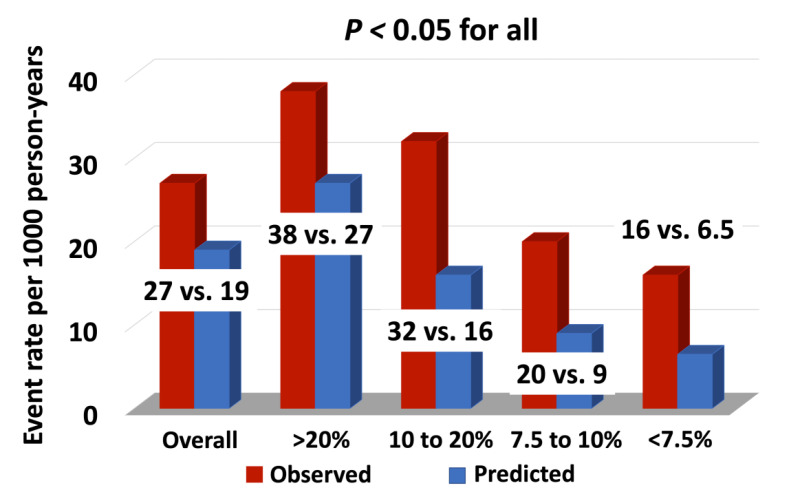
Current standard clinical calculators used for statin eligibility determination underestimate the risk for an ASCVD event. The bars represent predicted risk for an ASCVD event overall and in groups separated by <7.5% to over 20%. The red bars on the left represent observed risk for an ASCVD event overall and in groups separated by <7.5% to over 20%. The blue bars represent the average predicted risk in the group. ASCVD = Atherosclerotic Cardiovascular Disease.

We also calculated the predicted ASCVD rate per 1000 person-years using the standard ASCVD risk calculator and compared that with the observed ASCVD events per 1000 person-years, categorizing the cohort into ASCVD events by standardized cut-offs. These standardized cut-offs included groups separated by an estimated risk of <7.5% to over 20%. The overall observed ASCVD rate per 1000 person-years was 27 compared to the predicted rate of 19, translating into a 68% higher rate than expected (RR of 1.68, *p* < 0.001). Further, for an ASCVD rate per 1000 person-years for 10-year ASCVD risk >20%, observed vs. predicted (38 vs. 27%), for 10 to 20% (32 vs. 16%), for 7.5 to 10% (20 vs. 9%), and for <7.5% (16 vs. 6.5%), respectively: *p* < 0.05 (Figure 5).

We also compared the patients on statin treatment (288) to the ones not on statin treatment (723) for ASCVD events. The crude event rate was 15.7% among patients on statin vs. 17% who were not on statin (HR: 0.88, (95% CI: 0.71, 1.08, *p* = 0.24). After adjusting for age, sex, and cardiac risk factors, statin use was associated with a strong trend toward reducing ASCVD events (HR: 0.82, (95% CI: 0.68, 1.04); Table 8 and Table 9).

**Figure 6:**
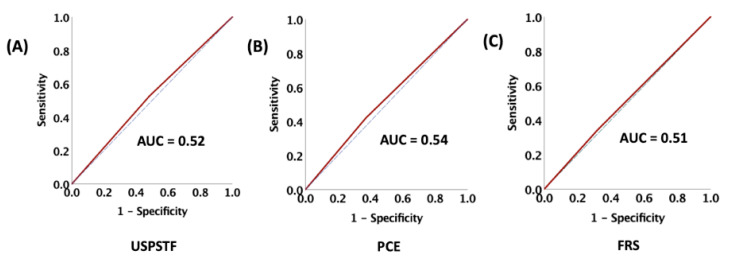
Assessment and comparison of the 3 model: (**A**) USPSTF, (**B**) PCE, and (**C**) FRS performances as an instrument to predict ASCVD events. ASCVD = Atherosclerotic Cardiovascular Disease, AUC = area under the curve.

Finally, to assess and compare the 3 model performances—USPSTF, PCE, and FRS—to predict ASCVD events, we generated the receiver operating curves (ROC). The areas under the curve (AUC) for all three models had poor values as instruments for predicting ASCVD events among HNCA patients receiving RT, as shown in Figure 6.

## 4. Discussion

In this study, we present data on the role of the major standard CV risk prediction models, including the ACC/AHA PCE, USPSTF, and FRS, in predicting long-term ASCVD risk among patients with head and neck cancers treated with RT. The observed rate of ASCVD was nearly 70% higher than that predicted following RT. Our data demonstrated that these risk scores do not reliably differentiate between those who will and those who will not have an ASCVD event after RT. Even in those patients predicted to have the lowest risk of ASCVD, an event rate of 10% or more was observed in follow-up, an event rate that would traditionally be considered high-risk.

The association between RT and ASCVD is well-established [3,18,19]. For example, studies among patients with Hodgkin’s lymphoma treated with RT have demonstrates a relative risk of 3 for cardiac death and of 5.5 for acute MI [20,21]. Similarly, an early Breast Cancer Trialists’ Collaborative Group analysis found that the risk of death from coronary heart disease increased by three percent per Gray (Gy) increase in the mean radiation dose [18]; moreover, a population-based study of more than 2000 women in Denmark and Sweden by Darby et al., noted that coronary events increased by 7.4% per Gy of the mean RT dose regardless of the presence of cardiac risk factors [18]. Among patients with HNCA, RT to the neck has been linked to accelerated carotid atherosclerosis. For example, in a meta-analysis that included 8 case-control studies and randomized clinical trials, Bashar et al. demonstrated that the incidence of extra-cranial carotid artery stenosis was 4–7-fold higher among the patients who received RT for HNCA compared to those with HNCA who did not receive RT [22]. This accelerated atherosclerosis with RT for HNCA is associated with ASCVD events. For example, Van Aken et al., in a prospective study involving 750 HNCA patients, showed an independent dose–effect relationship between RT dose to the neck and ischemic cerebrovascular events (HR = 1.11, AUC = 0.68) [23]. In our study cohort the overall ASCVD event rate per 1000 person-years was also high at 27 events per 1000-person years, with 18% of patients having an ASCVD event, complementary to the previously published data [20,21,24].

There may be value to expanding the indications for statin therapy among patients with HNCA treated with RT as data have suggested beneficial effects of statin therapy on reducing the risk of vascular events among patients receiving RT to the head and neck. For example, in a study involving patients with HNCA who were receiving RT, we previously showed a nearly 60% reduction in ischemic stroke and TIA and a >50% relative risk reduction in the development of ischemic stroke alone [24]. In another study involving 5718 patients with RT to the head, neck, and thorax, there was a 15% risk reduction in the primary outcome including cerebrovascular and cardiovascular events and 32% relative risk reduction in ischemic stroke alone among those on statins [25]. Similarly, in a retrospective study involving 15 vascular surgery or interventional radiology centers in France, Favre et al. demonstrated that statins were associated with a reduction in re-stenosis after carotid stenting in patients who developed symptomatic carotid stenosis after RT involving the neck [26]. The mechanism behind the favorable effects of statin against ASCVD with RT is probably mediated, in part, via an attenuation in the inflammatory process leading to reversal or stabilization in the atherosclerotic plaque burden [27,28,29,30,31]. This is supported by complementary pre-clinical data supporting a beneficial effect of statins for the risk of ASCVD post-RT to the head and neck. Specifically, in vitro data evaluating the effect of pravastatin on RT-treated endothelial cells showed statin use was associated with reduction in inflammation [32].

In 2016, the USTSPF released recommendations on statin use for the primary prevention of ASCVD. In these guidelines, individuals aged 40–75 years with 1 or more major ASCVD risk factors (hypertension, smoking, diabetes, or dyslipidemia) and a 10-year ASCVD risk equal of greater than 10% assessed by PCE were advised to initiate statin therapy as a primary prevention [11]. Similarly, the ACC/AHA guidelines use a PCE risk-based approach for statin therapy prescription for primary prevention, identifying a lower threshold for initiating statin therapy compared with USTSPF recommendations. The ACC/AHA recommended statin for individuals with a 10-year ASCVD risk greater than or equal to 7.5% using the PCE calculator without requirement for the presence of 1 major ASCVD risk factor [14]. The FRS is similar to the PCE; however, it is designed to estimate CAD alone and does not predict other significant atherosclerotic outcomes, such as CVA [15]. There are no wide-spread risk prediction models for ASCVD events that incorporate cancer/cancer therapy as a major effect modifier. Our data show that there was no difference in the incidence of ASCVD events between the statin eligible and non-eligible patients based on the ACC/AHA, USPSTF, and FRS recommendations suggesting that the PCE and FRS models lack precision post-RT for differentiating those at risk. The mechanisms for the underestimation of ASCVD risk post-RT is not clearly understood, with data suggesting a key role for inflammation and acceleration of atherosclerosis driven by endothelial cell injury and microvascular injury [33]. Furthermore, the pattern of plaque generation in the cardiovascular system appears to be different from that caused by conventional atherosclerosis [34]. For this reason, these ASCVD risk prediction models may underestimate risk among patients who have received RT, especially for HNCA.

### Study Limitations

This study needs to be interpreted within the context of the study design. The study was performed in a large cancer center, where study populations may differ from other community or suburban hospitals. The sample size is large and, while most of the patients were followed at our institution, we do not have access to the information on the ASCVD events that may have occurred in a different network, which would likely further increase ASCVD rates. We also could not adjust for cancer-related or cancer treatment–related factors such as RT dose and such unmeasured confounders may subsequently impact cardiovascular disease. However, our cohort had the same type of cancer with likely a similar treatment protocol, and it seems unlikely that eligibility for primary prevention with a statin would have impacted the RT dose. Survivor bias and loss of follow-up may have influenced the prevalence of statin eligibility and incident ASCVD risk in our cohort. Similarly, given that the risk of ASCVD was less known during the early experience with RT, adverse events may have gone uncaptured despite extensive search.

## 5. Conclusions

RT-treated patients face a significantly elevated risk of ASCVD, even after accounting for traditional baseline risk factors. Our data suggest that routinely available risk prediction tools do not adequately capture the risk for these events. Given the expected and continued increase in RT use, further studies characterizing the timing, mechanisms, and prevention of ASCVD after RT are needed and randomized studies testing the role of statin therapy in patients with HNCA receiving RT considered to be at lower traditional risk are warranted. Additionally, it may be reasonable to consider more aggressive approaches, such as targeting lower-predicted ASCVD risk thresholds and developing better risk prediction tools in patients with HNCA who have been treated with RT. Since this data mainly focuses on HNCA, further studies of patients receiving RT for other cancer types are warranted.

## Figures and Tables

**Table 1:** Demographics—Comparison of statin eligible vs. statin non-eligible groups based on PCE criteria.

Variables	Total Cohort	Statin Eligible	Statin Non-Eligible	*p*-Value
	*n* = 723 *	*n* = 359 *	*n* = 347 *	
Male	506 (70%)	248 (69%)	245 (71%)	0.68
Age	60 (51, 68)	60 (52, 69)	60 (51,68)	0.42
BMI	27 ± 5.6	27.5 ± 5.6	27.0 ± 5.8	0.24
Diabetes Mellitus	151 (21%)	142 (38%)	0 (0%)	<0.001
Hypertension	318 (44%)	188 (52%)	118 (34%)	<0.001
Hyperlipidemia	204 (28%)	148 (41%)	52 (15%)	<0.001
PVD	28 (3.9%)	14 (3.8%)	0 (0%)	<0.001
Carotid artery disease	17 (2.4%)	9 (2.5%)	8 (2.3%)	1.00
TIA	18 (2.5%)	10 (2.8%)	7 (2.1%)	0.63
CHF	30 (4.1%)	18 (5%)	11 (3.3%)	0.26
Atrial fibrillation	31 (4.2%)	14 (3.8%)	15 (4.3%)	0.85
Current/past smoker	325 (45%)	168 (47%)	143 (41%)	0.13
Systolic blood pressure	127 ± 19.0	127.5 ± 17.7	126.0 ± 20.2	0.29
Diastolic blood pressure	75 ± 10.6	75 ± 10.4	75 ± 10.8	1.00
Cardiac medications				
Aspirin	177 (24.5%)	89 (25%)	76 (22%)	0.37
Beta-blocker	176 (24.3%)	90 (25%)	78 (22%)	0.43
ACE I/ARB	179 (24.7%)	88 (24.5%)	90 (26%)	0.67
Cancer medications				
Platinum	460 (63.6%)	233 (65%)	222 (64%)	0.81
Anthracycline	33 (4.5%)	16 (4.5%)	15 (4.3%)	1.00
5FU	64 (8.9%)	20 (5.5%)	31 (8.7%)	0.11
Taxol	296 (41.0%)	147 (41%)	149 (43%)	0.59

* Numbers represent those with available data. In this table, 17 patients had incomplete data to calculate the PCE. Abbreviations: ACE I = Angiotensin-converting enzyme inhibitors, ARB = Angiotensin II receptor blockers, BMI = Body mass index, CHF = Congestive heart failure, PCE = Pooled Cohort equation, PVD = Peripheral vascular disease, TIA = Transient ischemic attack, 5FU = 5-Fluorouracil.

**Table 2:** Demographics—Comparison of statin eligible vs. statin non-eligible groups based on USPSTF criteria.

Variables	Total Cohort	Statin Eligible	Statin Non-Eligible	*p*-Value
	*n* = 723 *	*n* = 274 *	*n* = 423 *	
Male	506 (70%)	197 (72%)	296 (70%)	0.61
Age	60 (51, 68)	61 (51, 69)	60 (51, 68)	0.43
BMI	27 ± 5.6	26.8 ± 5.2	27.4 ± 5.7	0.16
Diabetes Mellitus	151 (21%)	136 (48%)	0 (0%)	<0.001
Hypertension	318 (44%)	168 (61%)	133 (32%)	<0.001
Hyperlipidemia	204 (28%)	142 (52%)	51 (12%)	<0.001
PVD	28 (3.9%)	9 (3.2%)	0 (0%)	<0.001
Carotid artery disease	17 (2.4%)	8 (2.9%)	8 (2.1%)	0.44
TIA	18 (2.5%)	8 (3%)	9 (2.2%)	0.62
CHF	30 (4.1%)	11 (3.9%)	14 (3.3%)	0.68
Atrial fibrillation	31 (4.2%)	11 (3.9%)	15 (3.4%)	0.84
Current/past smoker	325 (45%)	113 (41.5%)	179 (42.3%)	0.81
Systolic blood pressure	127 ± 19.0	127.5 ± 18.6	126 ± 19	0.31
Diastolic blood pressure	75 ± 10.6	75 ± 11	75 ± 10.4	1.00
Cardiac medications				
Aspirin	177 (24.5%)	75 (27.5%)	93 (22%)	0.12
Beta-blocker	176 (24.3%)	71 (26%)	95 (22.5%)	0.32
ACE I/ARB	179 (24.7%)	63 (23%)	105 (25%)	0.65
Cancer medications				
Platinum	460 (63.6%)	169 (62%)	271 (64%)	0.57
Anthracycline	33 (4.5%)	14 (5.2%)	18 (4.3%)	0.58
5FU	64 (8.9%)	25 (9.3%)	38 (9.1%)	1.00
Taxol	296 (41.0%)	110 (40%)	178 (42%)	0.64

* Numbers represent those with available data. In this table, 26 patients had unavailable data required for the USPSTF. Abbreviations: ACE I = Angiotensin-converting enzyme inhibitors, ARB = Angiotensin II receptor blockers, BMI = Body mass index, CHF = Congestive heart failure, PVD = Peripheral vascular disease, TIA = Transient ischemic attack, USPSTF = United States Preventive services Task Force, 5FU = 5-Fluorouracil.

**Table 3:** Demographics—Comparison of statin eligible vs. statin non-eligible groups based on FRS.

Variables	Total Cohort	Statin Eligible	Statin Non-Eligible	*p*-Value
	*n* = 723 *	*n* = 234 *	*n* = 455 *	
Male	506 (70%)	156 (67%)	327 (72%)	0.16
Age	60 (51, 68)	60 (52, 69)	60 (50,68)	0.42
BMI	27 ± 5.6	27.4 ± 5.9	27.2 ± 5.6	0.66
Diabetes	151 (21%)	119 (49%)	0 (0%)	<0.001
Hypertension	318 (44%)	147 (62%)	152 (33%)	<0.001
Hyperlipidemia	204 (28%)	117 (50%)	59 (14%)	<0.001
PAD	28 (3.9%)	10 (4.5%)	0 (0%)	<0.001
Carotid artery disease	17 (2.4%)	8 (3.6%)	9 (2.0%)	0.30
TIA	18 (2.5%)	6 (2.7%)	10 (2.2%)	0.79
CHF	30 (4.1%)	11 (4.6%)	17 (3.8%)	0.55
Atrial fibrillation	31 (4.2%)	11 (4.6%)	16 (3.6%)	0.53
Current/past smoker	325 (45%)	101 (43%)	213 (47%)	0.38
Systolic blood pressure	127 ± 19.0	127.3 ± 17.2	126.0 ± 19.6	0.39
Diastolic blood pressure	75 ± 10.6	75 ± 10.8	75 ± 10.2	1.00
Cardiac medications				
Aspirin	177 (24.5%)	68 (29%)	99 (22%)	0.04
Beta-blocker	176 (24.3%)	65 (28%)	105 (23%)	0.19
ACE I/ARB	179 (24.7%)	69 (29%)	114 (25%)	0.24
Cancer medications				
Platinum	460 (63.6%)	154 (66%)	282 (62%)	0.36
Anthracycline	33 (4.5%)	16 (6.7%)	20 (4.5%)	0.21
5FU	64 (8.9%)	20 (8.5%)	42 (9.2%)	0.89
Taxol	296 (41.0%)	89 (38%)	191 (42%)	0.33

* Numbers represent those with available data. In this table, 34 had unavailable data for FRS calculation. Abbreviations: ACE I = Angiotensin-converting enzyme inhibitors, ARB = Angiotensin II receptor blockers, BMI = Body mass index, CHF = Congestive heart failure, FRS = Framingham Risk Score, PVD = Peripheral vascular disease, TIA = Transient ischemic attack, 5FU = 5-Fluorouracil.

**Table 4:** Event rate per 1000 person-years.

ASCVD Event	Statin Eligible				Statin Non-Eligible			
	Rate	No. of Pts	No. of Events	Pts w/Events	Rate	No. of Pts	No. of Events	Pts w/Events
				**USPSTF**				
Total	28	274	54	44 (16%)	26	423	75	59 (14%)
CVA	11	274	22	16 (5.8%)	10	423	31	21 (5.2%)
MI	6	274	12	8 (2.9%)	5	423	14	8 (1.8%)
CHD	5	274	10	10 (3.6%)	6	423	16	16 (3.6%)
Cardiac Death	5	274	10	10 (3.6%)	5	423	14	14 (3.4%)
**PCE**								
Total	27	359	69	54 (15%)	25	347	59	46 (13.5%)
CVA	11	359	28	18 (5%)	10	347	23	14 (4%)
MI	5	359	13	8 (2.2%)	5	347	12	8 (2.3%)
CHD	6	359	15	15 (4.2%)	5	347	12	12 (3.4%)
Cardiac Death	5	359	13	13 (3.6%)	5	347	12	12 (3.4%)
**FRS**								
Total	29	234	45	36 (15.5%)	27	455	78	62 (13.7%)
CVA	10	234	16	11 (4.7%)	9	455	26	17 (3.8%)
MI	7	234	11	7 (3%)	6	455	17	10 (2.4%)
CHD	7	234	11	11 (4.7%)	7	455	20	20 (4.6%)
Cardiac Death	5	234	7	7 (3%)	5	455	15	15 (3%)

Some patients have more than one event, hence the total number of events is > total number of pts with events. Abbreviations: CHD = coronary heart disease, CVA = cerebrovascular accident, FRS = Framingham Risk Score, MI = myocardial infarction (Non-fatal), PCE = Pooled Cohort Equation, Pts w/events = patients with events, USPSTF = United States Preventive Services Task Force.

**Table 5:** Cox regression analysis, outcome—ASCVD event.

ASCVD Criteria	Hazard Ratio	95% CI		*p*-Value
		Lower	Upper	
USPSTF	1.36	0.82	1.62	0.26
PCE	1.28	0.86	1.72	0.32
FRS	1.17	0.76	1.58	0.42

Abbreviations: ASCVD = Atherosclerotic Cardiovascular Disease, FRS = Framingham Risk Score, PCE = Pooled Cohort Equation, USPSTF = United States Preventive services Task Force.

**Table 6:** Competing risk model (Fine–Grey Model), outcome—ASCVD event.

ASCVD Criteria	Hazard Ratio	95% CI		*p*-Value
		Lower	Upper	
USPSTF	1.31	0.69	1.52	0.27
PCE	1.20	0.72	1.64	0.31
FRS	1.12	0.68	1.48	0.38

Abbreviations: ASCVD = Atherosclerotic Cardiovascular Disease, FRS = Framingham Risk Score, PCE = Pooled Cohort Equation, USPSTF = United States Preventive Services Task Force.

**Table 7:** Poisson regression analysis, outcome—ASCVD event.

ASCVD Criteria	IRR	95% CI		*p*-Value
		Lower	Upper	
USPSTF	1.15	0.84	1.65	0.36
ACC/AHA	1.17	0.87	1.57	0.38
FRS	1.16	0.74	1.87	0.47

Abbreviations: ASCVD = Atherosclerotic Cardiovascular Disease, CI = Confidence Interval, FRS = Framingham Risk Score, IRR = incidence rate ratio, PCE = Pooled Cohort Equation, USPSTF = United States Preventive Services Task Force.

**Table 8:** **ASCVD** event rate per 1000 person-years, by statin status.

Patients Prescribed a Statin *n* = 288			Patients Not Prescribed a Statin *n* = 723		
Rate	No. of Events	Pts w/Events	Rate	No. of Events	Pts w/Events
23	53	45 (15.5%)	27	139	123 (17%)

Some patients have more than one event; hence, total number of events is > total number of pts with events. Abbreviations: ASCVD = Atherosclerotic Cardiovascular Disease, Pts = patients.

**Table 9:** Cox regression analysis of patients prescribed statin vs. those not prescribed statin; outcome—ASCVD event.

Model Type	Hazard Ratio	95% CI		*p*-Value
		Lower	Upper	
Unadjusted	0.88	0.71	1.08	0.24
Adjusted	0.82	0.68	1.04	0.28

The numbers are adjusted for age, sex, and cardiovascular risk factors. Abbreviations: ASCVD = Atherosclerotic Cardiovascular Disease, CI = confidence interval, Pts w/events = patients with events.

## Data Availability

The de-identified data underlying this article will be shared on reasonable request to the corresponding author with Institutional IRB approval.

## Abbreviations

ASCVD = atherosclerotic cardiovascular disease; USPSTF = United States Preventative Services Taskforce; HNCA = head and neck cancer; RT = radiation therapy.
